# 48 h for femur fracture treatment: are we choosing the wrong quality index?

**DOI:** 10.1186/s10195-019-0518-2

**Published:** 2019-02-13

**Authors:** Alessandro Aprato, Alessandro Casiraghi, Giovanni Pesenti, Marco Bechis, Alessandro Samuelly, Claudio Galante, Dario Capitani, Alessandro Massè

**Affiliations:** 1 School of Medicine, University of Turin, Viale 25 aprile 137 int 6, 10133 Turin, Italy; 2 Niguarda Ca’ granda Hospital, Milan, Italy

**Keywords:** Femur fracture, Early treatment, Walking

## Abstract

**Background:**

In the last 10 years, the rate of femur fractures treated within 48 h from trauma has been introduced as a performance index for hospital management in Italy. Literature showed a significant indirect correlation between early treatment and mortality/comorbidity. The aims of early treatment are pain management and reduction of time to ambulation. The purpose of this study is to evaluate whether early treatment has reduced time to ambulation in femur fracture.

**Materials and methods:**

All patients admitted to two level I trauma centers with proximal femoral fracture between 1/1/2017 and 31/12/2017 were included in this study. Exclusion criteria were patient age younger than 65 years, death before surgery, and nonsurgical treatment. The following data were collected: age, gender, date and time of admission to emergency department, height, weight, body mass index (BMI), type and side of fracture, American Society of Anesthesiologists (ASA) score, date and time of surgery, surgical time, length of hospitalization, death during hospitalization, time from surgery to physiotherapy start, and time from surgery to first walking day.

**Results:**

The study sample resulted in 660 patients. Mean age was 82 years, 64 % were female, mean BMI was 24 kg/m^2^, mean ASA score was 2.7, and 42 % were medial fractures. Mean time from admission to surgery was 95 h; 49.8 % were treated within the first 48 h. Mean time from surgery to physiotherapy start was 2 days, 21 % were not able to walk during hospitalization, time from surgery to first walking day was 5 days, and mean hospitalization time was 15 days. Early surgery was significantly (*p* = 0.008) associated with the probability of ambulation recovery during hospitalization. No association (*p* = 0.513) was found between early surgery and time in bed without walking.

**Conclusions:**

Early surgery in femur fracture became a priority in the health system. However, according to our data, although 51 % of patients were treated within the first 48 h, time from surgery to physiotherapy start (2 days) was still too long. Furthermore, time from surgery to first walking day was 6 days, longer than in most published papers. These data suggest that the performance index (rate of femur fractures treated within 48 h) may be improved by changing it to rate of femur fractures surgically treated with return to walking in 96 h.

**Level of evidence:**

Level 4 (retrospective study).

## Introduction

Hip fractures in the elderly will increase greatly in the coming decades due to aging of the population [1, 2]. Evidence suggests that surgery is the most effective treatment for femur fracture, and recent guidelines assess that early surgical treatment reduces mortality and complications [3–7]. Several published papers have suggested a cutoff of 48 h for operation [5]. The aims of early treatment are pain management and to reduce time to ambulation. Since 2008, the Ministry of Health in Italy has introduced the rate of femur fracture treated within 48 h as one of the indicators of hospital efficiency [8]. The aim of this study is to evaluate whether early treatment reduced time to ambulation in femur fracture.

## Materials and methods

### Settings

All patients admitted to two level I trauma centers with proximal femoral fracture between 1/1/2017 and 31/12/2017 were included in this study. Exclusion criteria included patient age younger than 65 years, death before surgery, and nonsurgical treatment.

### Data collection

Hospital charts were retrospectively reviewed after patients had given informed consent for use of their data. The present study was approved by the Institutional Review Board and was conducted in accordance with the ethical standards laid down in the 1964 Declaration of Helsinki and its later amendments. The following data were collected: age at admission, gender, date and time of admission to emergency department (ER), height, weight, body mass index (BMI) [9], type of fracture (pertrochanteric, subtrochanteric, basicervical, subcapital, transcervical; then grouped into intracapsular or extracapsular), side of fracture (right or left), American Society of Anesthesiologists (ASA) score [10], date and time of surgery, surgical time, length of hospitalization, death during hospitalization, time from surgery to physiotherapy start, time from surgery to first walking day, and time from physiotherapy start to first ambulation (including and excluding weekends and public holidays).

Patients were also divided into two groups according to early (within 48 h) or delayed (> 48 h) surgical treatment in order to evaluate whether early surgery (among other factors) was significantly related to early recovery of ambulation and to time from surgery to first walking day. All data were analyzed using standard descriptive statistics. The data for the two trauma centers were compared using the chi-squared test or Fisher’s exact test for categorical outcomes, and Student’s *t* test or Mann–Whitney test for continuous outcomes. The Kolmogorov–Smirnov test was used to determine whether data were normally distributed. *p*-Values lower than 0.05 were considered statistically significant. All analyses were performed using Stata version 12 (Stata Corporation, College Station, TX, USA).

## Results

Out of 785 patients (636 from trauma center I, 149 from trauma center II), 125 were excluded (71 younger than 65 years, 48 nonsurgical treatment, 3 death before surgery, and 3 incomplete data). Therefore, the study sample resulted in 660 patients (516 from trauma center I, 144 from trauma center II). Table 1 presents baseline data, while Table 2 presents timing, ability to walk before discharge, and mortality.. Among patients who walked again after surgery, 54 % were operated within 48 h from admission, while 46 % were operated after 48 h. Among patients who did not walked again after surgery, 41 % were operated within 48 h from admission, while 59 % were operated after 48 h. The data show a decreasing trend in reaching ambulation among patients operated after 48 h from admission, with a statistically significant association (*p* = 0.008, Pearson correlation coefficient).

**Table 1:** Baseline data

Age (years)	84 (78.8–88.0)
Women (%)	74.8
BMI (kg/m^2^)	23.4 (20.8–26.4)
ASA score	3.2
Type of fracture (%)	
Basicervical	18
Subtrochanteric	13
Pertrochanteric	42
Subcapital	18
Transcervical	1
Type of surgery (%)	
Intramedullary nail	53.4
Other devices	4
Hemiarthroplasty	34
Total arthroplasty	8
Mean surgical time	74 min (SE 1.16 min)

SE, standard error

**Table 2:** Timing, ability to walk before discharge, and mortality for patients at both trauma centers

Mean time between admission and surgery	48.4 h (IQR 13–78 h)
Patients treated within 48 h from admission	49.80%
Patients able to walk before discharge	471 (78.2%)
Died during hospitalization	2.10%
Mean duration of hospitalization	12 days (IQR 9–16 days)
Mean time from surgery to physiotherapy start	2 days (IQR 1–3 days)
Mean time from surgery to first walking day	5 days (IQR 4–7 days)

IQR, interquartile range

Patients who underwent operation within 48 h from admission started to walk again after 5.4 days on average, versus 5.9 days for patients operated later than 48 h from admission, albeit without a statistically significant association (*p* = 0.0658, Mann–Whitney test).

Comparing the two trauma centers (Table 3), no significant differences emerged regarding demographic data (age, sex, BMI, ASA score). Even if there were significant differences in favor of trauma center I in terms of length of hospitalization (*p* = 0.001) and the rate of patients treated in the first 48 h (*p* = 0.002), there were no differences considering the time between surgery and start of physiotherapy (*p* = 0.838), time between surgery and start of ambulation (*p* = 0.846), or probability of walking again after surgery (*p* = 0.185).

**Table 3:** Comparison between the two trauma centers

	Trauma center I	Trauma center II	*p*-value
Number of patients	516	144	
Age (years)	83.6 (75.3–89.9)	83.9 (76.3–91.5)	0.150
Female (%)	74.2	77	0.485
Male (%)	25.8	23	
BMI (kg/m^2^)	23.7 (19.5–27.9)	23.6 (18.7–28.5)	0.878
Fracture type (%)			
Basicervical	8.8	8.3	
Mediocervical	16.7	18.7	0.037
Subtrochanteric	58.5	47.3	
Pertrochanteric	16	25.7	
Medial fracture (%)	42.7	52.7	0.032
ASA 1–2 (%)	47.4	36.2	0.215
ASA 3–5 (%)	52.6	63.8	
Treatment within 48 h (%)	53	39	*0.002*
Time from surgery to physiotherapy start (days)	2.39 (0.15–4.63)	2.32 (0.10–4.54)	0.838
Time from surgery to first walking day (days)	5.94 (2.39–9.49)	5.81 (2.02–9.6)	0.846
Able to walk during hospitalization (%)	77.3	83.5	0.185
Hospitalization time (days)	13 (5.9–20.1)	18 (2.4–33.6)	*0.001*

Italic values indicate significance of *p* value (*p* < 0.05)

## Discussion

The main purpose of this study is to evaluate whether early surgery of femoral fracture influences early recovery of ambulation in elderly people.

The data, like in other studies [11], show that early surgery significantly increases the probability of ambulation recovery but is not significantly correlated with faster recovery of ambulation.

This finding may be explained by poor attention to a fast and effective rehabilitation protocol: our study shows that recovery of ambulation takes significantly longer than the average results found in literature. In our study, patients experienced an average of 5.2 days of immobility, while in the majority of studies found in literature the average is 2 days [12–14], with only the study by Siu et al. [15] showing results comparable to ours. More importantly, our study did not show any significant differences between the two trauma centers in terms of either time between surgery and start of physiotherapy or time between surgery and start of ambulation.

Even if there is no clear and structured protocol to improve mobility after surgery for femoral fracture (Cochrane Review, Handoll [16], AAOS), numerous international guidelines (NICE [17], SIGN [18], NZGG, SEGG-SECOT, and GEIOS [19]) recommend that mobilization start on the day of the operation or the day after. A prospective study by Koval et al. [20] investigated the effects of immediate unrestricted weight bearing after femoral fracture; the results supported its application, since it was not associated with an increase of comorbidities.

The results of another prospective study by Siu et al. [15] showed that early ambulation is associated with a better outcome in terms of 6-month mortality, with a greater benefit for more vulnerable patients. The effect of immobility was mediated largely by postoperative delay. Furthermore, in a trial by Marcantonio et al. [21], early ambulation was associated with lower incidence of delirium, along with other determining factors.

Comparison of our results with existing literature highlights how early recovery of ambulation depends mostly on hospital protocols for rehabilitation and how early ambulation could lead to a reduction of hospitalization time and fewer nosocomial comorbidities [22, 23]. Our study provides an interesting analysis on this topic, based on data from a significant number of patients coming from two major trauma centers. Based on these results and the analogies between the two centers, it can be asserted that the question of early recovery of ambulation plays an important role not only at the local or regional scale, but presumably at the national scale. Consequently, this study highlights the importance of and necessity for appropriate and efficient organization of rehabilitation [24], which can lead to a great reduction of the duration of hospitalization and a smaller number of nosocomial comorbidities.

This study is limited by its retrospective design; furthermore, only patients and hospitals from a single European country were included, thus findings may not be generalizable to other geographical regions. Another limitation is the absence of data regarding the walking ability of the patients before trauma. Also, this study lacks clinical follow-up to understand whether its conclusions are also supported by the mortality rate.

Our results suggest that, even if early surgery increases the probability of walking again after femur fracture, it is not the only factor influencing the time between surgery and first walking day. Our data suggest that, although 51 % of patients were operated within 48 h, both the time between surgery and start of physiotherapy (median 2 days) and the time between surgery and recovery of ambulation (5–6 days) were above expectations and above the average results published in literature (2–3 days). Nonmedical issues largely influenced the time from surgery to physiotherapy start and from surgery to first walking day. In conclusion, we think that a direct connection between physiotherapist and orthopedic communities should be established to reduce these timings, and the performance indicator of the rate of fractures operated within 48 h should be improved, possibly by converting it to the rate of patients who are operated and start to walk again within 96 h.

## Abbreviations

ER: Emergency department
BMI: Body mass index
ASA: American Society of Anesthesiologists (ASA)
